# Leveraging machine learning to develop a postoperative predictive model for postoperative urinary retention following lumbar spine surgery

**DOI:** 10.3389/fneur.2024.1386802

**Published:** 2024-06-26

**Authors:** Samuel L. Malnik, Ken Porche, Yusuf Mehkri, Sijia Yue, Carolina B. Maciel, Brandon P. Lucke-Wold, Steven A. Robicsek, Matthew Decker, Katharina M. Busl

**Affiliations:** ^1^Department of Neurosurgery, Barrow Neurological Institute, St. Joseph's Hospital and Medical Center, Phoenix, AZ, United States; ^2^Lillian S. Wells Department of Neurosurgery, University of Florida, Gainesville, FL, United States; ^3^Department of Biostatistics, University of Florida, Gainesville, FL, United States; ^4^Departments of Neurology and Neurosurgery, University of Florida, Gainesville, FL, United States; ^5^Department of Anesthesiology, University of Florida, Gainesville, FL, United States

**Keywords:** lumbar surgery, machine learning, postoperative complications, risk factors, urinary catheterization, urinary retention

## Abstract

**Introduction:**

Postoperative urinary retention (POUR) is the inability to urinate after a surgical procedure despite having a full bladder. It is a common complication following lumbar spine surgery which has been extensively linked to increased patient morbidity and hospital costs. This study hopes to development and validate a predictive model for POUR following lumbar spine surgery using patient demographics, surgical and anesthesia variables.

**Methods:**

This is a retrospective observational cohort study of 903 patients who underwent lumbar spine surgery over the period of June 2017 to June 2019 in a tertiary academic medical center. Four hundred and nineteen variables were collected including patient demographics, ICD-10 codes, and intraoperative factors. Least absolute shrinkage and selection operation (LASSO) regression and logistic regression models were compared. A decision tree model was fitted to the optimal model to classify each patient’s risk of developing POUR as high, intermediate, or low risk. Predictive performance of POUR was assessed by area under the receiver operating characteristic curve (AUC-ROC).

**Results:**

903 patients were included with average age 60 ± 15 years, body mass index of 30.5 ± 6.4 kg/m^2^, 476 (53%) male, 785 (87%) white, 446 (49%) involving fusions, with average 2.1 ± 2.0 levels. The incidence of POUR was 235 (26%) with 63 (7%) requiring indwelling catheter placement. A decision tree was constructed with an accuracy of 87.8%.

**Conclusion:**

We present a highly accurate and easy to implement decision tree model which predicts POUR following lumbar spine surgery using preoperative and intraoperative variables.

## 1 Introduction

Postoperative urinary retention (POUR) refers to a patient’s inability to completely empty their distended bladder following surgery. POUR is a common complication across all surgical specialties with an incidence of 5%–70% (1, 2). Following spine surgery, average rates of POUR range from 5 to 38% depending on the definition of POUR, study population, and surgical characteristics (1, 3–8). The occurrence of POUR leads to discomfort and the potential need for catheterization, factors that overtly impact patient well-being. POUR has also been extensively linked to increased risk for serious complications such as urinary tract infection, sepsis, increased length of stay, higher medical costs, and increased rates of readmission to the hospital (4, 5, 9–11). In addition to immediate patient well-being and comfort, POUR was found to lower patient satisfaction, with patients who experienced POUR being less likely to be satisfied with spine surgery even at long-term follow up (11).

Several patient specific risk factors have been associated with the development of POUR following lumbar spine surgery with age and male sex being the most frequently described factors (4, 5, 9, 10, 12). Likewise, numerous surgical factors such as operative time, number of operative levels, and fusion/surgical instrumentation have been associated with POUR (4, 5, 11–13). While dozens of factors have been analyzed, few of these analyses have brought forth actionable plans for identifying patients at greatest risk for POUR outside of single variable analysis. These univariate approaches fail to adequately analyze the complex interactions of patient and surgical variables which limits their predictive accuracy.

Machine learning has become widely popularized in the spine surgery literature over the past decade with its application being put forward toward diagnosis of spinal conditions and prediction of surgical complications and outcomes (14). Previously, our group published a highly accurate model using preoperative variables to predict POUR through regression and neural network analysis (15); however, it did not account for intraoperative and perioperative variables during anesthesia, such as administration of narcotics, that have been demonstrated to affect a patient’s likelihood to develop POUR (16–18). Herein, we present a machine learning comprehensive approach for identification and classification of patients at risk for POUR following lumbar spine surgery with patient, surgical and anesthesia variables. We hypothesize that the inclusion of a greater spectrum of variables will increase the fidelity of the predictive model. Practically, this would enable the surgical team to better identify patients at greatest risk for POUR, proactively adjust expectations, and arrange for proper monitoring and mitigating strategies.

## 2 Methods

### 2.1 Study design

We performed a retrospective review of consecutive patients who underwent spine surgery at our tertiary care academic medical center from June 2017 to June 2019. Patients were identified for inclusion in the database by query of CPT codes specific to lumbar spine operations: 22533, 22534, 22558, 22585, 22612, 22614, 22630, 22633, 22634, 63005, 63012, 63017, 63030, 63035, 63042, 63047, 63048, 63056, 63057. Patients were excluded if surgery was not done through the clinic setting, had surgery in a non-lumbar region (i.e., thoracic, or cervical level), or were <18 years old. Study design and data security methods were approved by our Institutional Review Board under protocol #201902403.

### 2.2 Identification of variables

The data were retrospectively collected from charted demographic information, nursing and anesthesia reports, and neurosurgical operative reports. Preoperative variables included age, body mass index (BMI), and pre-surgical use of opioids or urinary retention medication (i.e., 5-alpha reductase inhibitors and/or alpha inhibitors). International Classification of Diseases (ICD) codes preexisting the surgical visit were collected from electronic health record (EHR) as well as Epic’s Care Everywhere® feature, a network connecting UF Health’s EHR to hundreds of other EHRs utilizing the Epic system (Epic Systems Corporation, Verona, Wisconsin). Intraoperative and post-operative variables were chosen based on previous studies and clinical suspicion of relevance (1, 9, 11–13, 15, 17–22). Intraoperative surgical variables included duration of surgery, indwelling catheter use, type of surgery (discectomy, laminectomy, and/or fusion), type of fusion if relevant, pelvic screw placement, number of levels, use of minimally invasive techniques, and surgical approach. Intraoperative anesthesia variables included total intravenous fluid administration, total volume of blood products transfused, and all medications administered during the surgical procedure.

### 2.3 Definition of POUR

Patients were monitored in the neuroscience intensive care unit, post-anesthesia care unit, and neurosurgical floor unit for failure to void and distended or painful bladders. Indwelling urinary catheters were placed intraoperatively for cases with expected surgery duration exceeding 3 h. In the absence of indwelling catheters, urine volume was determined per standard of care with nurse-led bladder scanning. POUR was defined as the reinsertion of indwelling urinary catheter, or the need for straight catheterization for urine volumes exceeding 400 mL on bladder scan (23, 24). Bladder scan was done with ultrasound in standard fashion. Timing of postoperative removal of the indwelling urinary catheter occurred at the discretion of the surgeon.

## 3 Statistical analysis

### 3.1 Variable selection

Four hundred and nineteen variables were collected including patient characteristics, ICD-10 codes, and intraoperative factors. Only patients with complete data sets were included in the analysis. To set up a model for predicting POUR, variables were selected in two steps. In the first selection stage, all variables were subjected to univariate analysis to reveal patterns of association with POUR. Mann–Whitney U-tests were used for continuous and nominal variables while chi-square tests were used for categorical variables. Following this analysis, variables were selected depending on statistical significance and refined based on previous literature (2, 15). Then, a LASSO regression approach based on a penalized regression to obtain shrinkage estimators where only variables that did not shrink to 0 were kept.

The data were randomly split into training (80%) and validation sets (20%). The training set was used to develop models to predict POUR. The validation set was used to evaluate the performance of the prediction models that fitted from the training data.

### 3.2 Predictive modeling

In building the predictive models, a logistic regression model is first fitted to predict POUR using the selected variables. The area under the curve (AUC) on both training and validation dataset was assessed to show the performance. Then, the predicted probability of having POUR for all patients from training and validation set is calculated from the logistic regression model. Based on the distribution of outcomes found in prior modeling based on pre-operative risk factors, we defined the top 11% of the predicted probability as high risk, the 74% as intermediate risk and the last 15% as low risk (15). Using the risk levels as outcome, a decision tree model is fitted to classify each patient’s risk level in the training set. Five-fold cross validation is utilized for hyper parameter tuning on minimum split and maximum depth. The accuracy of the decision tree is calculated from the validation set for performance evaluation. Brier score (measure of the accuracy of the probalistic prediction) was used to compare the forecasting ability of each aspect of the model, where the lower the score, the better the predictions are calibrated (25). All statistical analyses were performed using SAS statistical software.

## 4 Results

### 4.1 Clinical characteristics

**Figure 1:**
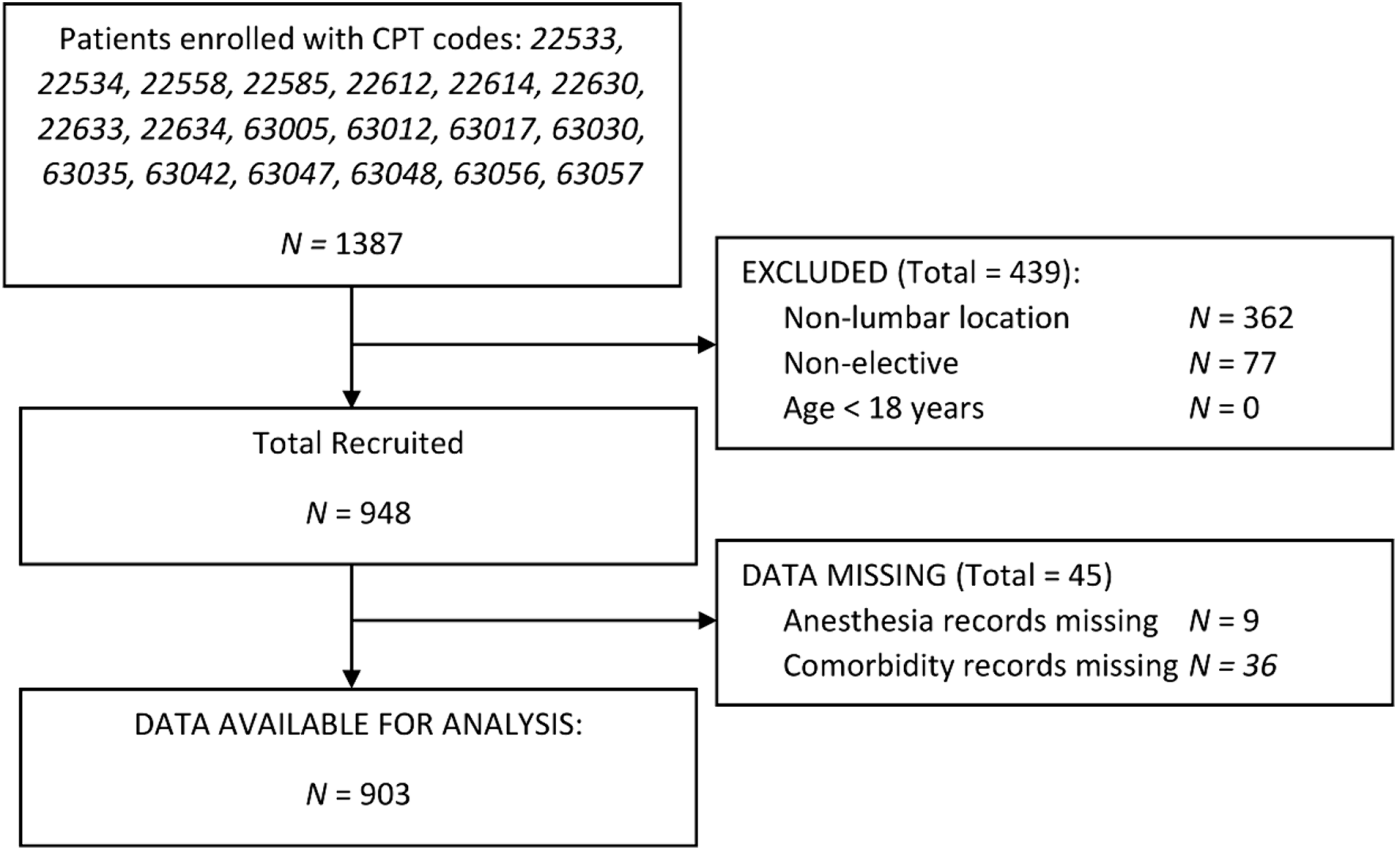
Flow diagram. CPT, Current procedural terminology.

**Figure 2:**
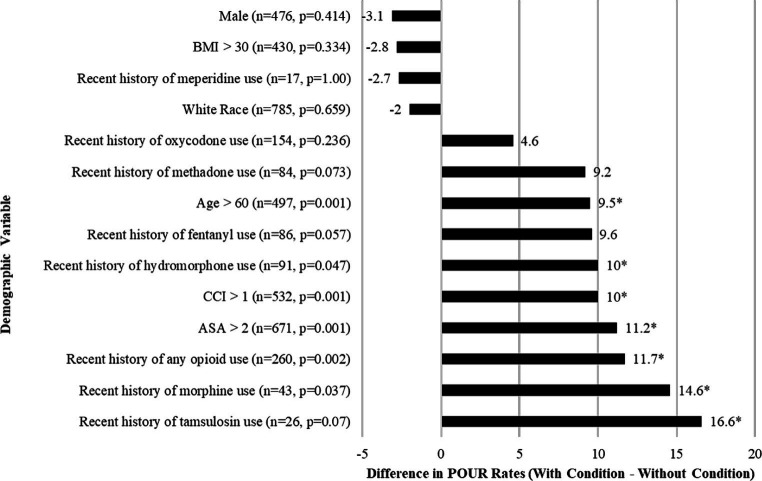
Bar graph of the differences in the rates of POUR based on preoperative clinical characteristics for patients who underwent lumbar spine surgery. Frequency (n) and *p*-values comparing those who did and did not develop POUR. Asterisk (*) indicates *p* < 0.05 in chi-square tests. BMI, Body mass index.

Of the 1,387 patients enrolled via CPT codes, 362 were non-lumbar, 77 were found to be non-elective, and 45 patients had missing data as shown in Figure 1. Of 903 patients included in this study, the mean age was 59.5 ± 15.4 years, BMI of 30.5 ± 6.4 kg/m^2^, 476 (53%) male, and 785 (87%) white. 24/903 (2.7%) had a history of UTI, and 27/903 (2.9%) had a history of retention. The incidence of POUR was 235 (26.1%) with 63 (7%) requiring indwelling urinary catheter placement. Patients who developed POUR were significantly older (62.2 ± 15.4 years vs. 58.5 ± 15.4 years, *p* = 0.002) but did not significantly differ with regards to BMI (30.6 ± 6.63 kg/m^2^ vs. 30.3 ± 5.85 kg/m^2^, *p* = 0.488), male sex (44.9% vs. 48.0%, *p* = 0.414), or white race (86.1% vs. 87.3%, *p* = 0.659). Differences in the rates of POUR based on preoperative clinical characteristics are shown in Figure 2. Patients who developed POUR were statistically more likely to have taken tamsulosin (+16.6%, *p* = 0.050) or opioids prior to surgery (+11.7%, *p* < 0.002), had an American Society of Anesthesiologist Physical Status Classification System (ASA) score > 2 (+11.2%, *p* = 0.001), and had a Charlson Comorbidity Index (CCI) > 1 (+10%, *p* = 0.001).

### 4.2 Surgical characteristics

**Figure 3:**
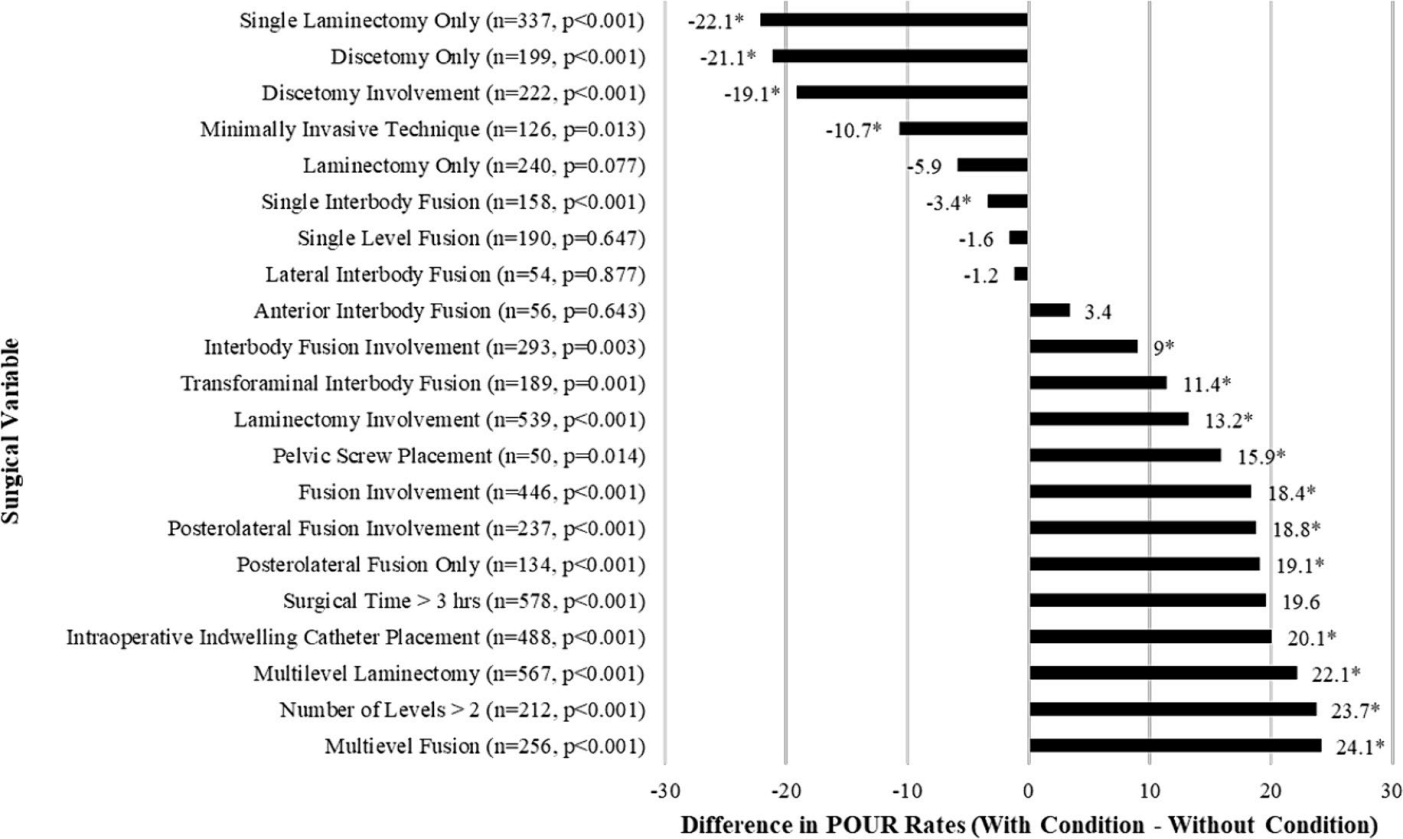
Bar graph of the differences in the rates of POUR based on categorical surgical variables for patients who underwent lumbar spine surgery. Frequency (n) and *p*-values comparing those who did and did not develop POUR. Asterisk (*) indicates *p* < 0.05 in chi-square test.

The differences in rates of POUR based on surgical variables are shown in Figure 3. There were multiple significant surgical predictors of POUR. Rates of POUR were significantly higher in patients with surgeries involving fusion (+18.4%, *p* < 0.001) or laminectomy (+13.2%, *p* < 0.001). The rates of POUR in patients who underwent multilevel laminectomy (+22.1%, *p* < 0.001) and multilevel fusion (+24.1%, *p* < 0.001) were higher. Intraoperative indwelling urinary catheter placement (+20.1%, *p* < 0.001) was a strong predictor of POUR. Similarly, there was a significant difference in the likelihood to develop POUR in patients who underwent surgery involving posterolateral fusion (+18.8%, *p* < 0.001), pelvic screw placement (+15.9%, *p* = 0.014) or interbody fusion (+9%, *p* < 0.003). Conversely, rates of POUR were significantly lower in patients whose surgery included discectomy only (−22.1%, *p* < 0.001) or involved discectomy (−19.1%, *p* < 0.001). Similarly, rates of POUR were significantly lower in patients who underwent minimally invasive technique operations (−10.7%, *p* < 0.001).

### 4.3 Anesthesia characteristics

A total of 69 variables were extracted and analyzed from intraoperative charts including muscle relaxants, reversal agents, vasopressors, antihypertensives, antibiotics, neuromuscular agents, sedatives, analgesics (opioids and non-opioid), intravenous fluids, and blood product transfusions. The average amount of 25 anesthesia variables were found to be significantly different between the groups of patients (Table 1). Patients who developed POUR had a significantly longer average surgical time (310 ± 147 min vs. 236 ± 130 min, *p* < 0.001), received greater volume of intravenous fluids (3,000 ± 2,330 mL vs. 1,960 ± 1,520 mL, *p* < 0.001), and received greater oral morphine equivalents (OME) of intravenous opioids (21.3 ± 35.0 mg OME vs. 13.1 ± 28.4 mg OME, *p* < 0.001).

**Table 1:** Selected anesthesia variables found to have statistically significant differences between the group of patients that developed POUR and the group of patients that did not develop POUR.

Variables—Mean (SD)^*^	POUR	No POUR	*p*-value	Correlation
Albumin (g)	17.7 (29.3)	7.5 (1.8)	<0.001	+
Calcium chloride	189 (711)	67.1 (342)	0.001	+
Calcium gluconate	189 (711)	71.5 (451)	0.014	+
Cefazolin	2,830 (2,060)	2,390 (1,720)	0.003	+
Dexamethasone	1.68 (3.43)	2.52 (3.98)	0.001	−
Ephedrine	12.2 (14.8)	9.18 (14.6)	0.005	+
Hydromorphone	0.397 (0.867)	0.262 (0.702)	0.031	+
Total IV Fluid Volume (mL)	3,000 (2,330)	1,960 (1,520)	<0.001	+
Ketorolac	1.91 (6.52)	4.49 (10.4)	<0.001	+
Methadone	3.96 (6.94)	2.41 (5.56)	0.001	+
Midazolam	0.536 (0.944)	0.805 (1.82)	0.003	−
Neostigmine	0.151 (0.784)	0.0419 (0.408)	0.043	+
Ondansetron	3.68 (1.27)	3.82 (1.17)	0.040	−
Oral Morphine Equivalents	21.3 (35.0)	13.1 (28.4)	0.001	+
Phenylephrine	6.39 (7.57)	2.99 (4.88)	<0.001	+
Plasma Transfusion (mL)	18.3 (134)	1.22 (23.8)	0.039	+
Plasmalyte (mL)	2,190 (1,480)	1,540 (1,210)	<0.001	+
Platelet Transfusion (mL)	6.94 (49.4)	0.753 (19.5)	0.038	+
Promethazine	0.0426 (0.460)	0.161 (1.15)	0.009	−
Propofol	948 (1,980)	645 (1,300)	0.019	+
RBC Transfusion (mL)	156 (546)	26.7 (167)	<0.001	+
Remifentanil	0.475 (1.40)	0.242 (1.35)	0.038	+
Rocuronium	87.0 (50.1)	75.9 (39.7)	0.006	+
Sufentanil	0.034 (0.068)	0.023 (0.045)	0.015	+
Surgery Time (min)	310 (147)	236 (130)	<0.001	+

Correlation indications relationship between variable and association with POUR. ^*^Units in mg unless otherwise mentioned. SD, Standard Deviation; POUR, Postoperative urinary retention; IV, Intravenous; RBC, Red Blood Cell.

**Figure 4:**
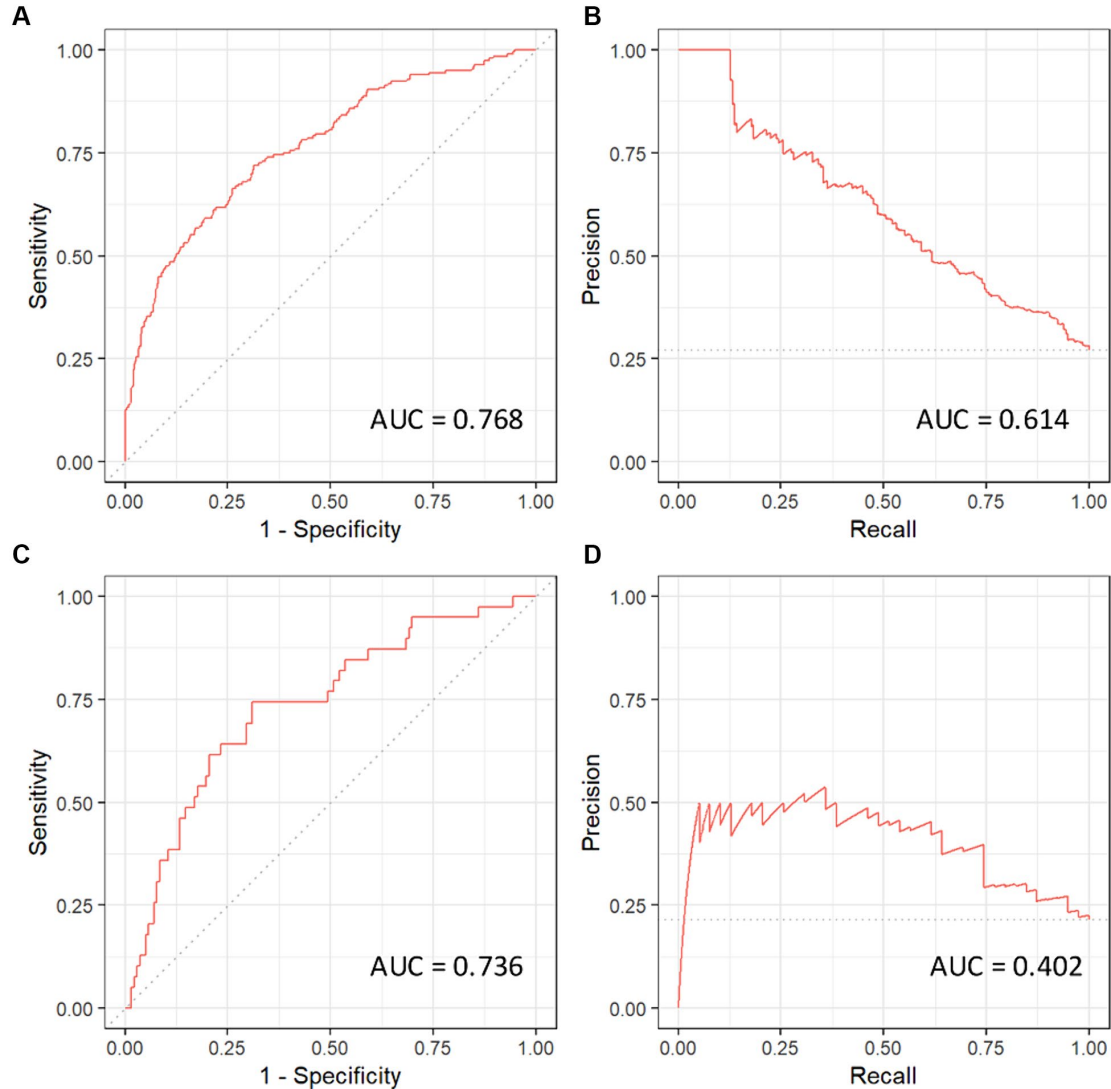
Receiver operating curves (ROC) and precision recall curves (PRC) for the logistic regression model. AUC, area under curve. **(A)** ROC-AUC for training set. **(B)** PRC-AUC for training set. **(C)** ROC-AUC for testing set. **(D)** PRC-AUC for testing set.

**Figure 5:**
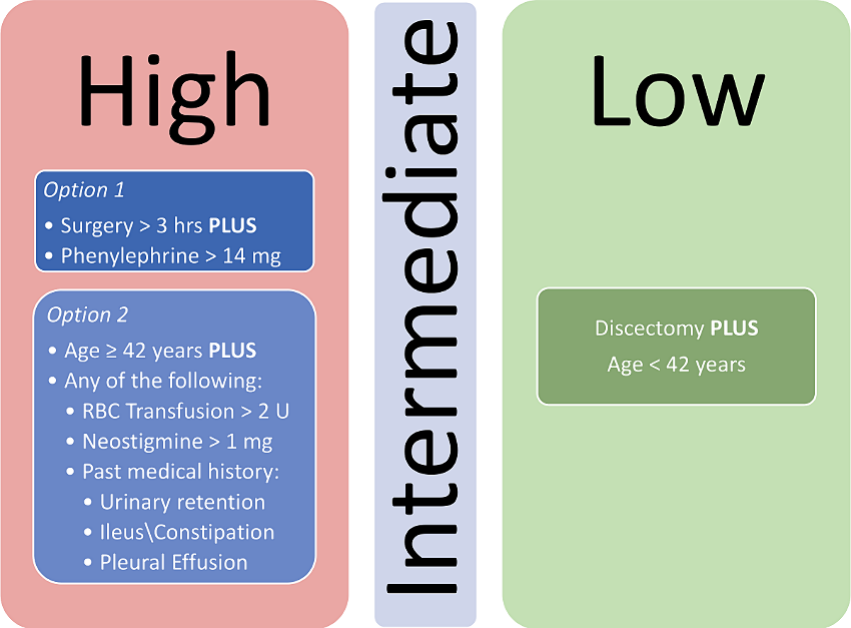
Decision tree model for stratifying patients for risk of POUR following lumbar spine surgery. RBC, Red blood cell.

Following initial univariate analysis of patient, surgical and anesthesia-related factors, 94 variables were selected for LASSO regression of which 13 variables did not shrink to 0. The LASSO regression model achieved an AUC of 0.676 on the testing set on the receiver operating characteristic (ROC) curve (training set AUC 0.743). The AUC on the precision recall curve (PRC) were 0.332 and 0.560 for the testing and training sets, respectively. After the model selection step, 14 variables including patient, surgical and anesthesia factors were isolated and included in logistic regression (Table 2). The logistic regression outperformed the LASSO regression model with an AUC-ROC of 0.737 (training set AUC 0.768; Figure 4). The AUC-PRC for this model on the testing and training sets were 0.614 and 0.402, respectively. After hyper-parametric tuning of selected predictors from the LASSO regression model, a decision tree model was constructed (Figure 5). The accuracy for the final decision tree model was confirmed to be 87.8% on a 3-class confusion matrix (which reduces to 70.9% on a confusion matrix excluding the intermediate category), with sensitivity 91.3%, specificity 55.2%, positive predictive value 61.0%, and negative predictive value 89.2%. Brier score was noted to be 0.19.

**Table 2:** Multivariate logistic regression analysis for the development of the POUR model.

Variable	Estimate	SE	Statistic	*p*-value
Age (years)	0.012	0.007	1.738	0.082
Arthrodesis—Z98.1	0.225	0.221	1.019	0.308
Cardiomegaly—I51.7	0.725	0.439	1.649	0.099
Constipation—K59.00	1.777	0.568	3.128	**0.002**
Discectomy Involvement	−0.389	0.317	−1.228	0.220
Ileus—K56.7	1.178	0.621	1.896	0.058
Intraoperative Foley	0.569	0.245	2.323	**0.020**
IV Fluid Volume (mL)	<0.001	<0.001	−0.579	0.562
Neostigmine (mg)	0.388	0.151	2.562	**0.010**
Number of Disc Levels	0.025	0.056	0.45	0.653
Phenylephrine (mg)	0.054	0.019	2.887	**0.004**
Pleural Effusion—J90	0.709	0.557	1.274	0.203
RBC Transfusion (mL)	0.001	<0.001	1.913	0.056
Retention of Urine—R33.9	2.621	0.678	3.866	**<0.001**

SE, Standard error of the coefficient; IV, Intravenous; RBC, Red Blood Cells.Bold values are statistically significant values defined as *p*-value < 0.05.

## 5 Discussion

POUR is an incompletely understood but frequently encountered barrier to patient recovery and satisfaction following lumbar spine surgery occurring in 25% of patients. Its pathogenesis is thought to be related to several factors including anesthetic agents, perioperative medications, and postoperative pain, all of which can alter the complex urinary signaling pathway. Anesthetics can act centrally at the pontine micturition center and peripherally as smooth muscle relaxants to decrease bladder contractility (26). Surgical pain or inadequate pain control further stimulates the sympathetic nervous system which acts to inhibit the detrusor muscle (27). Medications such as opioids are known to play dual functions by inhibiting parasympathetic and stimulating sympathetic innervations (28).

Thus far, no highly reliable and easily available prediction tools have been developed to identify *a priori* who is at increased risk for its development. Here, we present a model leveraging machine learning to classify the risk of a patient developing POUR following lumbar spine surgery using patient, surgical and anesthesia characteristics. Using machine learning, we were able to condense more than 90 variables associated with POUR in univariate analysis to a 14-variable logistic regression model and eventually constructed an eleven-node decision tree after hyper-parametric tuning of selected predictors from the LASSO regression model, with a final accuracy for the decision tree model of 87.8% on a confusion matrix and AUC-ROC of 0.737. This accuracy outperforms all previously available models and hence offers a novel and improved predictive tool for POUR.

The incidence of POUR within our study was 26% and is well within the incidence of POUR (5.6%–38%) reported across diverse studies of lumbar spine surgery (3, 5–7, 9, 12, 13). Previous studies have contained extensive inclusion and exclusion criteria for their models of POUR. We chose to include a heterogeneous patient population within our analysis to better understand how we can comprehensively evaluate the lumbar spine surgery population for the development of POUR. By utilizing the logistic and LASSO regression models, a decision tree was able to be constructed that outperforms any prior predictive tool with accuracy of 0.878.

### 5.1 Limitations and future aims

Our model has limitations. As with all algorithms, it is only as accurate as the data which it contains. In this case, it is derived from a large tertiary care referral center where comprehensive data about a patient’s past medical and surgical history may not be complete. We minimized this variability by extracting the medical history of patients from Epic’s Care Everywhere network (Epic Systems Corporation) which accesses patient’s medical charts from hundreds of other healthcare organizations, not exclusively our hospitals electronic medical record. Likewise, the study was retrospectively designed which carries biases inherent to a retrospective study.

This study was aimed at prediction of POUR, and not at interpretation of component variables. It serves as a diagnostic tool for POUR instead of identifying the critical variables that cause it. It can be tempting to elaborate on the meaning of predictors featured in the final model; however, these specific predictors are likely confounded by extensive patient and surgical variables and would warrant further prospective investigation. For factors such as phenylephrine (used for intraoperative blood pressure augmentation), a feasible alternative that is not associated with POUR, regardless of causality between the factor and POUR, might not exist. However, the use of intraoperative urinary catheters which appears to be statistically significant in all models, presents a potentially modifiable variable. While this variable is extensively confounded by surgical time and associated anesthesia requirements via medications and fluids, it remains important to investigate. Additionally, further improvement in the predictive capabilities of this model can be achieved by including baseline bladder/urologic functional status and preoperative urologic medication requirements.

## 6 Conclusion

In conclusion, we describe a highly accurate postoperative predictive model for POUR following lumbar spine using diverse preoperative and operative (surgical and anesthesia) variables. We were able to leverage machine learning to develop a 14 variable logistic regression model with an ROC-AUC of 0.737 and a decision tree model with an accuracy of 87.8%. These models substantially outperform previously published models of POUR in this patient population and include a greater spectrum of variables to highlight the effect of many less frequently appreciated variables. Furthermore, the final decision tree model is easy to implement clinically and can be put forth toward further studies aimed at preventing POUR following lumbar spine surgery. A prospective, multi-center study is needed to further validate our prediction model.

## Data availability statement

The original contributions presented in the study are included in the article/supplementary material, further inquiries can be directed to the corresponding author.

## Ethics statement

The studies involving humans were approved by the University of Florida Institutional Review Board. The studies were conducted in accordance with the local legislation and institutional requirements. Written informed consent for participation was not required from the participants or the participants’ legal guardians/next of kin in accordance with the national legislation and institutional requirements.

## Author contributions

SM: Methodology, Writing – original draft. KP: Conceptualization, Writing – review & editing. YM: Data curation, Resources, Writing – original draft. SY: Formal analysis, Visualization, Writing – original draft. CM: Conceptualization, Writing – review & editing. BL-W: Conceptualization, Formal analysis, Writing – review & editing. SR: Supervision, Writing – review & editing. MD: Project administration, Supervision, Writing – review & editing. KB: Project administration, Supervision, Writing – review & editing.

## References

[1] Baldini G Bagry H Aprikian A Carli F Warner DS Warner MA. Postoperative urinary retention: anesthetic and perioperative considerations. Anesthesiology. (2009) 110:1139–57. doi: 10.1097/ALN.0b013e31819f7aea 19352147

[2] Pomajzl AJ Siref LE. Post-op urinary retention. In: StatPearls. Treasure Island (FL): StatPearls publishing (2022). Available at: http://www.ncbi.nlm.nih.gov/books/NBK549844/ (Accessed 18 February 2022).

[3] Boulis NM Mian FS Rodriguez D Cho E Hoff JT. Urinary retention following routine neurosurgical spine procedures. Surg Neurol. (2001) 55:23–7. doi: 10.1016/S0090-3019(01)00331-7, PMID: 11248301

[4] Cremins M Vellanky S McCann G Mancini M Sanzari L Yannopoulos A. Considering healthcare value and associated risk factors with postoperative urinary retention after elective laminectomy. Spine J. (2020) 20:701–7. doi: 10.1016/j.spinee.2020.01.012, PMID: 32006710

[5] Gandhi SD Patel SA Maltenfort M Anderson DG Vaccaro AR Albert TJ. Patient and surgical factors associated with postoperative urinary retention after lumbar spine surgery. Spine. (2014) 39:1905–9. doi: 10.1097/BRS.0000000000000572, PMID: 25299169

[6] Jellish WS Thalji Z Stevenson K Shea J. A prospective randomized study comparing short-and intermediate-term perioperative outcome variables after spinal or general anesthesia for lumbar disk and laminectomy surgery. Anesth Analg. (1996) 83:559–64. doi: 10.1213/00000539-199609000-00021, PMID: 8780281

[7] Kopel J Sharma DP. Spinal surgery and urinary retention: a review of the literature. J Clin Urol. (2020) 14:4–9. doi: 10.1177/2051415820916932

[8] Porche K Samra R Melnick K Brennan M Vaziri S Seubert C. Enhanced recovery after surgery (ERAS) for open transforaminal lumbar interbody fusion: a retrospective propensity-matched cohort study. Spine J. (2021) 22:399–410. doi: 10.1016/j.spinee.2021.10.007, PMID: 34687905 PMC9595392

[9] Golubovsky JL Ilyas H Chen J Tanenbaum JE Mroz TE Steinmetz MP. Risk factors and associated complications for postoperative urinary retention after lumbar surgery for lumbar spinal stenosis. Spine J. (2018) 18:1533–9. doi: 10.1016/j.spinee.2018.01.022, PMID: 29447854

[10] Martinez OV Civetta JM Anderson K Roger S Murtha M Malinin TI. Bacteriuria in the catheterized surgical intensive care patient. Crit Care Med. (1986) 14:188–91. doi: 10.1097/00003246-198603000-00003 3943334

[11] Zakaria HM Lipphardt M Bazydlo M Xiao S Schultz L Chedid M. The preoperative risks and two-year sequelae of postoperative urinary retention: analysis of the Michigan spine surgery improvement collaborative (MSSIC). World Neurosurg. (2020) 133:e619–26. doi: 10.1016/j.wneu.2019.09.107, PMID: 31568914

[12] Altschul D Kobets A Nakhla J Jada A Nasser R Kinon MD. Postoperative urinary retention in patients undergoing elective spinal surgery. J Neurosurg Spine. (2017) 26:229–34. doi: 10.3171/2016.8.SPINE151371, PMID: 27767680

[13] Lee S Kim CH Chung CK Park SB Yang SH Kim SH. Risk factor analysis for postoperative urinary retention after surgery for degenerative lumbar spinal stenosis. Spine J. (2017) 17:469–77. doi: 10.1016/j.spinee.2016.03.017, PMID: 27012647

[14] DelSole EM Keck WL Patel AA. The state of machine learning in spine surgery: a systematic review. Clin Spine Surg. (2022) 35:80–9. doi: 10.1097/BSD.0000000000001208 34121074

[15] Porche K Maciel CB Lucke-Wold B Robicsek SA Chalouhi N Brennan M. Preoperative prediction of postoperative urinary retention in lumbar surgery: a comparison of regression to multilayer neural network. J Neurosurg Spine. (2021) 36:32–41. doi: 10.3171/2021.3.SPINE21189, PMID: 34507288 PMC9608355

[16] Sfeir S Mansour N. Post operative analgesia with intrathecal morphine. Middle East J Anaesthesiol. (2005) 18:133–9. PMID: 15830768

[17] Mormol JD Basques BA Harada GK Louie PK Alter K Goldberg E. Risk factors associated with development of urinary retention following posterior lumbar spinal fusion: special attention to the use of Glycopyrrolate in anesthesia reversal. Spine. (2021) 46:E133–8. doi: 10.1097/BRS.0000000000003678, PMID: 32890297

[18] Bowman JJ Edwards CC 2nd Dean C Park J Edwards CC Sr. Incidence and risk factors for postoperative urinary retention following lumbar spine fusion. Clin Spine Surg. (2021) 34:E397–402. doi: 10.1097/BSD.0000000000001202, PMID: 34050045

[19] Madani AH Aval HB Mokhtari G Nasseh H Esmaeili S Shakiba M. Effectiveness of tamsulosin in prevention of post-operative urinary retention: a randomized double-blind placebo-controlled study. Int Braz J Urol Off J Braz Soc Urol. (2014) 40:30–6. doi: 10.1590/S1677-5538.IBJU.2014.01.05, PMID: 24642148

[20] Aiyer SN Kumar A Shetty AP Kanna RM Rajasekaran S. Factors influencing postoperative urinary retention following elective posterior lumbar spine surgery: a prospective study. Asian Spine J. (2018) 12:1100–5. doi: 10.31616/asj.2018.12.6.1100, PMID: 30322244 PMC6284120

[21] Knight BA Bayne AP Zusman N Barney N Yang S. Postoperative management factors affect urinary retention following posterior spinal fusion for adolescent idiopathic scoliosis. Spine Deform. (2020) 8:703–9. doi: 10.1007/s43390-020-00090-9, PMID: 32077085

[22] Chang Y Chi K-Y Tai T-W Cheng YS Lee PH Huang CC. Risk factors for postoperative urinary retention following elective spine surgery: a meta-analysis. Spine J. (2021) 21:1802–11. doi: 10.1016/j.spinee.2021.05.009, PMID: 34015508

[23] Petros JG Bradley TM. Factors influencing postoperative urinary retention in patients undergoing surgery for benign anorectal disease. Am J Surg. (1990) 159:374–6. doi: 10.1016/S0002-9610(05)81274-7, PMID: 2316800

[24] Faas CL Acosta FJ Campbell MDR O'Hagan CE Newton SE Zagalaniczny K. The effects of spinal anesthesia vs epidural anesthesia on 3 potential postoperative complications: pain, urinary retention, and mobility following inguinal herniorrhaphy. AANA J. (2002) 70:441–7. PMID: 12526149

[25] Brier GW. Verification of Forecasts Expressed in Terms of Probability. Mon Weather Rev. (1950) 78:1–3. doi: 10.1175/1520-0493(1950)078<0001:VOFEIT>2.0.CO;2

[26] Kamphuis ET Ionescu TI Kuipers PW de Gier J van Venrooij GE Boon TA. Recovery of storage and emptying functions of the urinary bladder after spinal anesthesia with lidocaine and with bupivacaine in men. Anesthesiology. (1998) 88:310–6. doi: 10.1097/00000542-199802000-00007, PMID: 9477049

[27] Toyonaga T Matsushima M Sogawa N Jiang SF Matsumura N Shimojima Y. Postoperative urinary retention after surgery for benign anorectal disease: potential risk factors and strategy for prevention. Int J Color Dis. (2006) 21:676–82. doi: 10.1007/s00384-005-0077-2, PMID: 16552523

[28] Elsamra SE Ellsworth P. Effects of analgesic and anesthetic medications on lower urinary tract function. Urol Nurs. (2012) 32:60–8. doi: 10.7257/1053-816X.2012.32.2.60 22690461

